# Patient values informing medical treatment: a pilot community and advance care planning survey

**DOI:** 10.1136/bmjspcare-2016-001177

**Published:** 2017-03-02

**Authors:** S Milnes, C Corke, N R Orford, M Bailey, J Savulescu, D Wilkinson

**Affiliations:** 1 School of Medicine, Deakin University, Geelong, Victoria, Australia; 2 Intensive Care Unit, University Hospital Geelong, Barwon Health, Geelong, Victoria, Australia; 3 Department of Epidemiology and Preventive Medicine (DEPM), Australian and New Zealand Intensive Care Research Centre (ANZIC-RC), Monash University, Melbourne, Victoria, Australia; 4 Faculty of Philosophy, Oxford Uehiro Centre for Practical Ethics, University of Oxford, Oxford, UK; 5 John Radcliffe Hospital, Oxford, UK

**Keywords:** Clinical decisions, Quality of life, Values, Communication, Ethics, Advance Care Planning

## Abstract

Medicine regards the prevention of death as an important priority. Yet patients may have a range of priorities of equal or greater importance. These other priorities are often not discussed or appreciated by treating doctors.

**Objectives:**

We sought to identify priorities of care for patients attending an advance care planning (ACP) clinic and among the general population, and to identify factors associated with priorities other than prolonging life.

**Methods:**

We used a locally developed survey tool ‘What Matters Most’ to identify values. Choices presented were: maintaining dignity, avoiding pain and suffering, living as long as possible, and remaining independent. Participants rated the importance of each and then selected a main priority for their doctor. Participant groups were a purposive sample of 382 lay people from the general population and 100 attendees at an ACP clinic.

**Results:**

Living as long as possible was considered to be less important than other values for ACP patients and for the general population. Only 4% of ACP patients surveyed and 2.6% of our general population sample selected ‘living as long as possible’ as their top priority for medical treatment.

**Conclusions:**

‘Living as long as possible’ was not the most important value for ACP patients, or for a younger general population. Prioritisation of other goals appeared to be independent of extreme age or illness. When end of life treatment is being discussed with patients, priorities other than merely prolonging life should be considered.

## Introduction

A recent focus to identify ‘what matters’^1^ to patients when making goals of care decisions in health has led to consideration of what people value to live well.^2–4^ This is particularly important in people living with a life-limiting illness to ensure that their care is consistent with their values and goals.^4–7^ To help doctors and other health professionals participate in patient-centred, values-based discussions, it is important to identify how community-based populations consider and prioritise certain values for living well.

Decisions regarding patient care should include consideration of patient's values to inform goals of care.^8^ This is the basis for shared decision-making.^2^ ^9^ Unless patients communicate these values, doctors generally prioritise prolongation of life^10^ through care based on chance of cure or short-term benefit.^4^ ^11^ ^12^ In contrast, previous studies with patients suggest that important considerations in these decisions are maintaining dignity, relationships and independence.^4^ ^11^ Many doctors believe that discussion of alternative goals only becomes appropriate for those who are very elderly or in the final stages of disease.^13^

A number of studies have described goals of care relating to disease and treatment decisions, in community and patient cohorts.^11^ ^13^ ^14^ Other studies have explored doctors' decision-making based on patient values such as independence and longevity.^4^ ^9^ ^11^ ^12^ However, no studies have described the prioritisation of specific values to inform treatment decisions in a large community-based cohort.

The aims of this study were to identify how a community group and ACP client group rank and prioritise the four values of dignity, independence, freedom from pain and suffering, and longevity. We also sought to identify the effect of group, age and gender on the priorities expressed.

The purpose of the ACP clinic is to assist people from the community formulate an ACP with specially trained registered nurses. The clinic in this study is part of the larger health service and is free of charge with a referral from the primary or tertiary care services within the catchment area.

## Methods

### Design, ethics and consent

This study included two cohorts of participants who were asked to complete a survey designed to determine values. The first cohort comprised 100 consecutive patients attending a community ACP clinic in Geelong, a major regional city in South-Western Victoria, Australia. Sampling in this cohort was convenience based, with the first 100 attendees in the study period asked if they would participate in the paper-based survey. A plain language statement attached to the survey indicated that submission of the survey denoted consent. Participant demographics were similar to those of the usual attendees at the clinic.

The second cohort comprised a general community group, with medical doctors excluded. Recruitment in the second cohort occurred through purposive sampling of members of the general community using a snowball technique via social networks,^15^ ^16^ starting in Australia and the UK. Each of the authors sent a standardised email explaining the study to their family, friends and acquaintances who they thought might be interested in the content and were a cross mix of age groups. The email included a request for them to forward the email to people they believed would be interested in completing the survey. Further information was included on the first page of the survey tool once they entered the online site. A second round of emails was sent when the rate of response to the online survey slowed. It was made clear in the preamble of the online survey that completion and submission of the survey denoted consent. Since this was a pilot study, a sample size of 400 was chosen for convenience. Both the paper and online survey were non-identifiable. Ethics approval was obtained from the Barwon Health Research Governance and Integrity Unit.

### The survey

**Figure 1:**
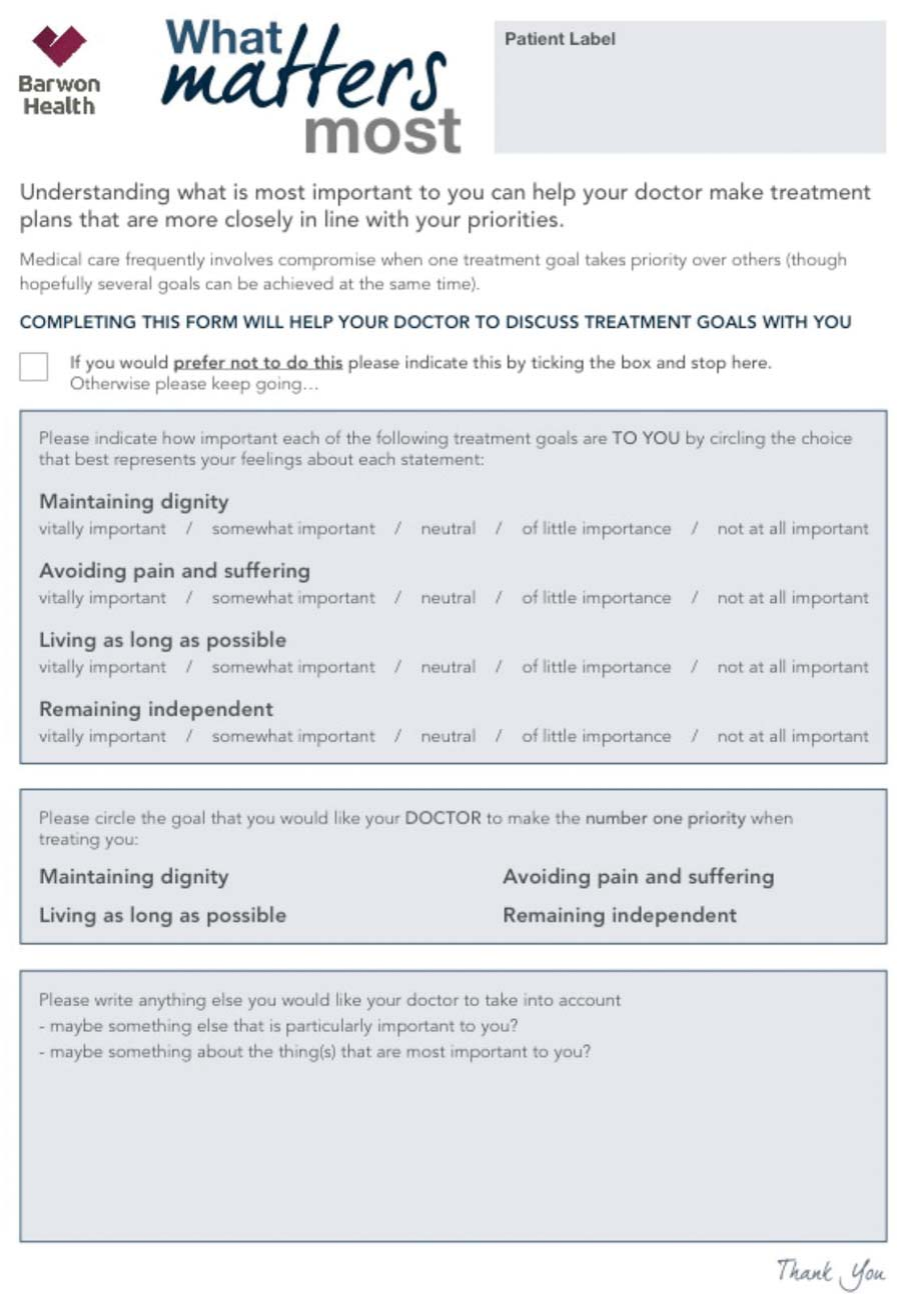
Information given to all participants both on-line survey and hard copy.

The ‘What Matters Most’ survey was created through a process of literature review of studies identifying values for living well and treatment decisions.^13^ ^14^ ^17^ Two authors (SM and CC) reviewed the literature and decided on the final four values represented in the survey. The ACP clinic participants received the ‘What Matters Most’ survey in paper form as they arrived for the ACP clinic appointment, and returned it to the facilitator on completion. The general community cohort conducted the survey using the SurveyMonkey survey tool (http://www.surveymonkey.com). Responses were recorded in a de-identified format. All information provided to participants is seen in figure 1.

Participants in both cohorts were asked to rate each of four values—maintaining dignity, remaining independent, avoiding pain and suffering, and living as long as possible—using a five-point Likert scale. The scale ranged from 1 (not important at all) to 5 (vitally important). Each value could be rated at any point of the scale. A second question asked participants to identify which value they would want their doctor to prioritise as a treatment goal for them. Demographic data including gender and age were also collected.

### Statistical analysis

Continuously normally distributed data were reported as mean (±SD), whereas non-parametric data were reported using median (IQR) or frequency distribution. Continuous variables were compared via Wilcoxon rank-sum test while binary variables were compared using Fisher's exact test. A p value <0.05 was considered significant. Likert scale responses were considered as ordinal data, assigned numbers 1–5, and compared between groups non-parametrically using median values. Where the median was uninformative, a mean was also provided.

## Results

A total of 100 participants were recruited into the ACP cohort, and 382 participants into the community cohort. The baseline demographics are presented in table 1. The response rate for the ACP group was 96%, with four of the patients handed the paper survey not returning it to the ACP consultant. These four attendees were not followed up and the sample size was considered final when 100 surveys were returned. We were unable to determine the response rate of the community cohort as that survey was online and only the results of those who ‘submitted’ were available. Also, it was not possible to include data from 18 participants in the community group due to incomplete data from the survey. The decision to remove these participants was to maximise representation of full data sets for matching priorities and ranking. The community cohort was significantly younger and had a larger proportion of female respondents.

**Table 1:** Characteristics of community and ACP clinic cohorts 2

	Community	ACP	p Value
Number	382	100	–
Female	287 (75.1)	63 (63.0)	0.02
Age (years)			
18–24	6 (1.5)	0 (0)	
25–34	42 (11.0)	0 (0)	
35–44	74 (19.4)	0 (0)	–
45–54	104 (27.2)	1 (1.0)	–
55–64	99 (25.9)	2 (2.0)	–
65–74	41 (10.7)	20 (20.0)	–
>74	16 (4.2)	77 (77.0)	–
Mean	50.5 (±13.5)	77.1 (±5.9)	<0.0001

Data are shown as median (IQR), number (%) or mean (±SD).

ACP, advance care plan.

The distribution of values by Likert scale scores for the community and ACP clinic patient cohorts are presented in table 2.

**Table 2:** Distribution of values between community and ACP clinic cohorts

	Community	ACP	p Value
Dignity			
No importance	1 (0.3)	1 (1.0)	
Not very important	3 (0.8)	0 (0)	
Neutral	7 (1.8)	4 (4.0)	
Some importance	84 (22.0)	21 (21.0)	
Very important	286 (75.0)	74 (74.0)	
Median	5 [5–5]	5 [4–5]	0.75
Mean	4.71 (±0.6)	4.67 (±0.7)	
Longevity			
No importance	43 (11.3)	16 (16.0)	
Not very important	71 (18.6)	19 (19.0)	
Neutral	145 (38.1)	24 (24.0)	
Some importance	93 (24.4)	22 (22.0)	
Very important	29 (7.6)	19 (19.0)	
Median	3 [2,4]	3 [2,4]	0.43
Mean	2.98 (±1.1)	3.09 (±1.4)	
Pain and suffering			
No importance	0 (0)	0 (0)	
Not very important	4 (1.1)	2 (2.0)	
Neutral	12 (3.2)	3 (3.0)	
Some importance	85 (22.4)	28 (28.0)	
Very important	279 (73.2)	67 (67.0)	
Median	5 [4,5]	5 [4,5]	
Mean	4.68 (±0.6)	4.6 (±0.7)	0.21
Independence			
No importance	1 (0.26)	1 (1.0)	
Not very important	6 (1.6)	1 (1.0)	
Neutral	20 (5.2)	5 (5.0)	
Some importance	150 (39.3)	22 (22.0)	
Very important	204 (53.5)	71 (71.0)	
Median	5 [4,5]	5 [4,5]	
Mean	4.44 (±0.7)	4.61 (±0.7)	0.004

Data are shown as number (%), median [IQR] or mean (±SD).

ACP, advance care plan.

Both cohorts rated highly dignity, avoidance of pain and suffering, and remaining independent (more than 90% regarding these values as somewhat important or vitally important) with longevity the lowest ranked value (30–35% regarded this as not important or not very important). These results were similar between groups with the exception of independence, which was rated significantly higher by the ACP group. When the cohorts were combined and compared by gender, a similar pattern was observed. Females rated dignity and independence significantly higher than males (table 3).

**Table 3:** Distribution of value preferences by gender for all participants

	Male		Female		p Value
Number	132		350		
Age	58.6 (±17.4)		54.9 (±15.9)		0.03
Dignity	5 [4,5]	4.54 (±0.7)	5 [5,5]	4.76 (±0.5)	<0.0001
Pain and suffering	5 [4,5]	4.59 (±0.6)	5 [4,5]	4.69 (±0.6)	0.06
Longevity	3 [2,4]	3.01 (±1.2)	3 [2,4]	3.01(±1.1)	0.97
Independence	5 [4,5]	4.37 (±0.8)	5 [4,5]	4.52 (±0.68)	0.05

Data are shown as number, median [IQR] or mean (+SD).

The distribution of value preferences was similar across all age ranges, with dignity, avoidance of pain and suffering, and remaining independent ranked highest, and longevity ranked lowest. The values of pain and suffering and independence varied significantly across age ranges, with the ranking of pain and suffering decreasing, and independence increasing in importance, with increasing age of respondents.

The values rated as most important are presented in table 4. The relief of pain and suffering was ranked as the most important value by the highest proportion of participants in the community and ACP cohorts, followed by maintaining dignity and remaining independent. Living as long as possible was ranked as most important by the lowest proportion of participants. Analysis by referral source, age and gender revealed no differences in ranking.

**Table 4:** Distribution of top priority value comparing community and ACP cohorts

	Referral source			Age								Gender		
Top priority	ACP	Community	p Value	18–24	25–34	35–44	45–54	55–64	65–74	>74	p Value	Male	Female	p Value
No	100	382	–	6	42	74	105	101	61	93	–	132	350	–
Dignity	29 (29.0)	107 (28.0)	0.85	1 (16.7)	8 (19.0)	23 (31.1)	28 (26.7)	30 (29.7)	19 (31.1)	27 (29.0)	0.81	33 (25.0)	103 (29.4)	0.33
Pain and suffering	35 (35.0)	170 (44.5)	0.09	3 (50.0)	23 (54.8)	34 (45.9)	47 (44.8)	42 (41.6)	22 (36.1)	34 (36.6)	0.46	63 (47.7)	142 (40.7)	0.16
Longevity	4 (4.0)	10 (2.6)	0.85	1 (16.7)	1 (2.4)	0 (0)	4 (3.8)	3 (2.9)	1 (1.6)	4 (4.3)	0.27	4 (3.0)	10 (28.6)	0.92
Independence	32 (32.0)	95 (24.9)	0.85	1 (16.7)	10 (23.8)	17 (22.9)	26 (24.8)	26 (25.7)	19 (31.1)	28 (30.1)	0.88	32 (24.2)	94 (26.9)	0.55

Data are shown as number (%).

ACP, advance care plan.

## Discussion

### Main findings

This study of two cohorts of adults, one community based and one referred to an advance care planning clinic, found that dignity, avoidance of pain and suffering, and remaining independent were the values considered most important by both groups, while longevity was least important. Older respondents placed less importance on avoidance of pain and suffering, and more importance on remaining independent. This age-related change in values could explain the difference between the ACP and community cohorts. Finally, the rating order of importance of values was the same for men and women, although there were significant differences in the rating of dignity and independence.

### Relationship to existing literature

Patient-centred care requires identification of patient's values and preferences or goals.^18^ Doctors' inertia to discuss preferences and goals of care with patients with life-limiting illness is well documented.^5^ ^17^ ^19^ ^20^ The inability to identify patient goals and values could result in failure to respect patient autonomy, confusion among the treating team, failure to articulate clear points of treatment direction and limitation, and the continuation of unwanted, expensive and invasive medical care.^21^ This study adds to the current literature by identifying a list of values that the general community, including community-based people with a life-limiting illness, consider important when identifying goals of care. Previous studies have concentrated on patients listing values they consider important in the context of their current health status. This keeps the discussion around the disease/illness context rather than on what matters to them outside hospital. In this study, the identification of specific values and their importance for the general community and community-based patients with a life-limiting illness may help doctors overcome barriers to communication and planning.^17^ ^19^ ^20^

Advance care planning focuses on identifying values for living well^8^ and associated goals of care and treatment limitation,^22^ in a patient population for whom hospitalisation and intensive medical treatment may not be beneficial. Often, these patients have a life-limiting illness and disease trajectory that changes with each hospital admission.^23^ The ACP cohort in our study rated freedom from pain and suffering lower than did the general population. This is important as when considering how to communicate with patients, as the current literature defines preferences in terms of risk/benefit analysis of outcomes, including pain and suffering, longevity, and disability,^11^ ^17^ ^24^ ^25^ there is evidence of a potential mismatch between how doctors and patients prioritise treatment decisions,^4^ ^10–12^ with doctors prioritising longevity. The finding that patients prioritise independence and dignity above both freedom from pain and suffering and longevity is an important addition to this area.

### Strengths and limitations

The strength of this study is that it provides doctors and healthcare workers involved in communication with patients with a starting point to identify values that matter to patients to live well. Doctors find it difficult to keep conversations patient-centred and are more comfortable discussing disease context.^3^ ^19^ ^20^ This list may help doctors to direct the conversation towards living well rather than immediate medical treatment.

We developed a simple, paper-based tool called ‘What Matters Most’. We included values of dignity, independence, freedom from pain and suffering, and longevity (living as long as possible). While this tool cannot be used to comprehensively infer specific treatment preferences, it may help initiate shared decision-making, and facilitate discussions between healthcare professionals and patients about values, goals of care, treatment alternatives and priorities.

Values identified by patients in the literature include maintenance of relationships, communication, independence, feeling worthwhile with a sense of purpose, freedom from pain and suffering and living with a level of disability.^4^ ^12^ ^13^ To keep the survey simple for our wider population, we simplified some of these to dignity and independence. This should be tested qualitatively on a broad population to verify a community understanding of each of these in the health or illness context.

The advance care population surveyed was from a single geographic area in Australia, and results may be different in other social, cultural or religious groups. While our general population sample yielded similar values to the ACP group, this purposive sample was not selected randomly, and may not be representative of the wider community. We did not collect data on participant religion or ethnic background. Our sample had a large percentage of female respondents, which may influence results (though we are not aware of evidence of a gender difference regarding willingness to have a discussion about values in the shared decision-making, communication and end-of-life literature). The use of purposive sampling of social contacts may have led to participants with similar value considerations to the researchers. Future research should assess values in a larger, heterogeneous community sample.

Finally, doctors were excluded from this study in an attempt to obtain a representation of lay persons' responses not influenced by professional health literacy. However, we did not exclude or identify other health professionals.

## Conclusion

Discussions of priorities of care with patients should be structured around what is important to them for living well. While it is necessary to consider likely medical outcomes for treatment decisions, doctors should ask patients about what they value. From this study, it is apparent that people across a wide age range value remaining independent and living with dignity while minimising pain and suffering over longevity.
